# A review of urban planning tools for empowering public participation

**DOI:** 10.1007/s44243-025-00067-0

**Published:** 2025-11-01

**Authors:** Samuel Hanan, Neil Carhart

**Affiliations:** Civil Engineering, University of Bristol, Queen’s Building, University Walk, Bristol, BS8 1TR Bristol UK

**Keywords:** Urban planning, Citizen participation, Software, GIS, Serious games

## Abstract

The ever-increasing proportion of humanity residing in cities has led to recognition of the rights and potential of citizens in the design of conurbations, but there remains a technical knowledge gap between communities and professional planners. This gap is exemplified in the software tools utilised in urban planning practice, yet these tools also have scope for empowering communities towards democratic and informed decision making. Given the recent proliferation of such tools, this paper evaluates a range of them, from established GIS software and rapid concept generation and analysis applications, to more novel methods emerging from interoperability platforms and the videogame industry, to help provide genuine agency to the public without compromising the quality of existing design processes. Each tool was tested through examination of its respective functions in typical industry scenarios according to a set of characteristics derived from relevant literature and ISO 25000 metrics, enabling assessment and comparison of their use cases. The research concludes that the gap between citizens and planners can be bridged through a combination of functionality from the software tools surveyed, including the computational power of GIS software, parametric modelling and generative design from rapid concept generation and analysis applications, software interoperability from interoperability platforms, and accessible user interfaces and detailed simulation from the videogame industry. Development of a workflow comprising Giraffe Urban Analytics, Speckle, and Cities Skylines II is suggested for further research, which is to be informed by the useful functionality detailed in this paper.

## Introduction: who designs the city?

Urban planning involves “management for very complex systems... necessarily multidimensional and multi-objective in its scope” (Hall 2002). Initial approaches to this intricate challenge consisted of a single visionary with a masterplan; the first of these in the UK being Ebenezer Howard with Garden Cities (Howard et al. 1965), followed by Sir Patrick Geddes with his single-iteration design method of survey, analysis, plan (Hall 2002). Le Corbusier, a European contemporary, then imagined the *City Radieuse* (Radiant City), but it remained only an idea. While his *Unité d’Habitation* did see construction, the total overhaul that his vision required never received sufficient political will (Hall 2002), and this gap between ideas and reality similarly plagued many Garden City implementations (Hall 2014). Robert Moses, working in 20^th^ century New York, was able to make his personal vision for the city a reality, but only through extensive political manoeuvring that rendered him the city’s sole urban planning decision maker without any election to office (Mars and Kalan 2024), with his anti-democratic methods since criticised (Caro 2015).

A contrasting approach to this top-down urban planning began emerging with Jane Jacobs’ 1961 book The Death and Life of Great American Cities (Jacobs 2000). She derisively made the point that planners cocooned in their offices did not understand why cities worked, but merely contributed preconceived ideas of how they ought to. Personally clashing with Robert Moses, she advocated mixed-use zoning over broad-stroke architectural plans and focused on tangible community rather than idealised human action. Combined with the limited and debatable success of previous masterplans, this resulted in a turning to citizens for their real understanding of where they lived. Their insight was officially acknowledged in the UK with the 1969 Skeffington Report, which required that public participation be part of the planning process (Hall 2002).

This inclusion of the public in planning practice addressed a challenge occurring simultaneously in planning theory. The emerging potential of computing power for demystifying the complexity of conurbations had led to development of the systems approach to planning. This methodologically identifies mathematical relationships that map onto the observed operations of cities in the hope that these can then forecast future growth so that the resulting demands can be met through a well-informed decision making process (Batty 1976; McLoughlin 1969; Chadwick 1978). Two key elements of this process are feedback loops and goal setting (Hall 2002). Feedback loops help address the intransigency of single-iteration plans, but acting upon feedback is only beneficial if set goals are actually desirable. This challenge is left unaddressed by additional computing power and myriad data-rich analyses; Jacobs’ fundamental issue of a lack of understanding on the part of planners remains. The insight of communities in setting auspicious goals is needed for systematic computer models to see their benefits unlocked, and current computational power is rarely invoked to aid them thus, even in the basic form of simple 3D visualisations (Eilola et al. 2023).

Communities possess the insight to make a significant contribution to urban planning (Zellner 2024; Lawson et al. 2022). The process by which their knowledge is integrated into the urban planning process is thus worthy of exploration.

## Literature review I: public consultation and the knowledge gap

**Fig. 1:**
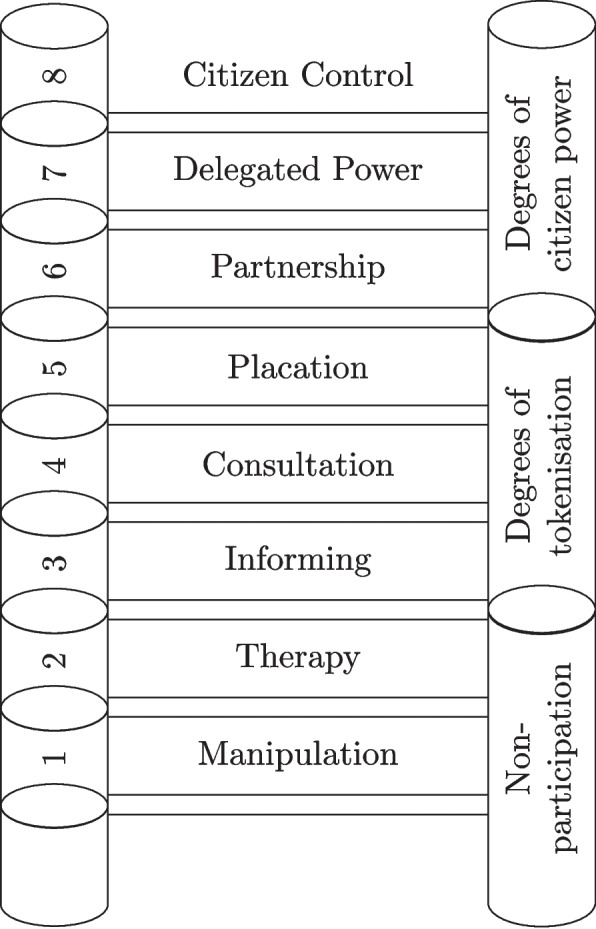
Arnstein’s ladder of citizen participation (Arnstein 1969, Fig. 2)

Multiple international frameworks now provide for citizen participation in urban planning (Mougiakou et al. 2023). However, the implementation of public consultation by decision makers is regularly reduced to a box-ticking exercise without any intention of implementing community views (Lawson et al. 2022; Kahila-Tani et al. 2019), not least because the problem is often decided upon before consultation begins (Cooney and Raghavan 2022). This box ticking appears prevalent enough that it has been incorporated into frameworks for measuring citizen participation. Arnstein’s Ladder of Citizen Participation (Arnstein 1969), a common such framework, categorises any measures taken into eight rungs as shown in Fig. 1. The first of these is Manipulation, where community members may be given roles with a pretence of responsibility but that serve only to gain their approval on designs they don’t understand so that developers can claim they were engaged. Consultation, the term more generally used when residents are included in the design process, is the fourth rung of ladder in a section summarised as “degrees of tokenism”. The top rung is Citizen Control, where communities have the authority, knowledge, and funding to deliver the changes they desire. This ranking thus argues that citizen are not really participating unless they are given the power to enact changes themselves, implying that many contemporary methods are merely tokenism.

Another method for measuring public participation is the International Association for Public Participation’s framework, which has five levels: Inform, Consult, Involve, Collaborate, and Empower (International Association for Public Participation 2018). Two literature reviews using this framework (Foroughi et al. 2023; Nasr-Azadani et al. 2022) found the majority of case studies in public participation were at the Consult level, with none reaching the Empower level, similarly suggesting that in reality, citizen participation is no more than a token gesture.

Citizen knowledge is often sidelined in favour of professional opinion because of the perceived expertise gap between the two. One sense of expert knowledge is “empirical knowledge on whether, how and with what strength certain actions have caused certain effects in various contexts” (Tennøy et al. 2016) - that is, knowledge attainable only through experience which the general public lack. However, whether this knowledge exists in reality is debated (Tennøy et al. 2016), as any implementation of it is too context-specific for empirical insights to be generalisable, and because cause-effect relations are challenging to ascertain in urban planning. This is why the computer models of the systems approach could not solve planning on their own: “[they] could not be mapped onto planning problems that were always too diverse, ill-defined and ambiguous to admit of highly structured decision-making supported by well-defined computer technologies” (Batty 2012). Further, a tendency for planners to apply “outdated knowledge or undocumented ‘planning myths’ ” (Tennøy et al. 2016) was identified, often arising as, once graduated from education, they learn from the examples and anecdotes of previous planners, rather than new academic literature.

Additional difficulties faced by current public participation practice include planners’ lack of knowledge of engagement methods, the tendency of these methods to reach only the same small group of time-rich participants, limitations in the quality of the information produced, and a lack of trust from the public in planners (Kahila-Tani et al. 2019), which can lead to a silent majority not engaging at all (Foroughi et al. 2023). These issues all derive from the larger problem that extensive technical knowledge is required to truly participate in planning (Alhassan 2025). This is another facet of expert knowledge which somewhat explains why community voices are given limited weight by decision makers: they are not trusted to have informed opinions due to their lack of academic and professional credentials. However, providing citizens a formal planning education is clearly impractical; challenges arise from “the great volumes of documentation and changing information involved in planning and placemaking” (Lawson et al. 2022), an amount of reading people simply do not have time for (Cooney and Raghavan 2022), and the technical language of this documentation limits those without a formal education by undermining their credibility in professional meetings which they attempt to join (Lawson et al. 2022). Further, there is the challenge of understanding relevant regulations. Citizen struggles have been observed on this front in England’s Neighbourhood Planning system, through which a community can develop a legally valid plan for the future development of their area. Their ideas are still subservient to preexisting local plans and must undergo external evaluation, and thus they “can continually and objectively fail the process, as their neighbourhood visions interact with the hard reality of statutory planning regulations” (Lawson et al. 2022).

It is the technical knowledge gap then, rather than the experiential one, that primarily hampers public participation in planning. One way in which this gap is exemplified is planners’ familiarity with professional software tools and models. They have a dedicated education on this front (Cooney and Raghavan 2022) where they work with models that are intelligible only to experts (Grêt-Regamey and Fagerholm 2024). These tools thus often requires too much technical knowledge for citizens to meaningfully interact with them (Vazquez et al. 2023; Najafi et al. 2023). However, software also has the potential to engage non-technical stakeholders (Nasr-Azadani et al. 2022) by reducing temporal and locational constraints on them (Conroy and Evans-Cowley 2006) and providing the information needed for citizens in terms of simplifying technical language and streamlining regulatory compliance (Vazquez et al. 2023). With human-computer interaction becoming ubiquitous (Salim and Haque 2015), system design incorporating it has given non-technical stakeholders access to interaction with complex processes (Fang et al. 2022) - though fully realising this potential remains a challenge, with the fundamental design principles being the critical factor (Lyu 2024).

## Literature review II: surveying the established field

In order to bridge the technical knowledge gap through software tools, the existing tools must first be surveyed to understand the current capabilities of the field. Any such survey needs a comprehensive range of both the number of tools analysed and the set of functions they are assessed against for a complete understanding. This is demonstrated by an examination (El Meouche et al. 2013) of the interaction between BIM and GIS software which evaluated tools based on the level of information retained through the import process from one to the other. This elucidates software interoperability as important functionality, but it is the only feature analysed among many on offer. Further, this research considered only a narrow range of tools; Autodesk Revit was the singular BIM tool, with Google Earth, AutoCAD Map 3D, and ArcGIS comprising the GIS offering.

A wider range of tools is present in the literature, with an analysis (Behmanesh and Brown 2019) of their occurrence within it including Esri CityEngine, Modelur, CityPlanner, Space Syntax (DepthmapX and QGIS), Urban Modelling Interface, ArcGIS, and UrbanFootprint finding ArcGIS to be the most commonly cited in academic work. This selection was then analysed according to their data collection, data analysis, modelling, and visualisation capabilities.

An alternative approach (Al-Douri 2018) directly questioned planning practitioners as to their everyday experience of using various tools and functionalities. It found untapped potential in dynamic visualisation (including virtual reality), simulating pedestrians, and what-if planning scenarios. It was suggested that these were limited due to the ability and experience of users with the tools, and because of their typical position within the design process: they are brought in to articulate ideas, not to create them. Another questionnaire (ul Hussnain et al. 2020) examined reasons for the limited adoption of digital planning tools, finding their functionality lacking in available urban data, workflow integration, and user friendliness.

Reviews in slightly different contexts identified further functionality that remains applicable to urban planning. One (O’Connell et al. 2023) discusses the benefits of several GIS tools for marine renewable energy, evaluating them according to their included data, included calculations, and user interface design. Another (Palomino et al. 2017) examined thirty-one GIS tools on their support for collaboration, specifically discussing their interoperability and APIs, and whether they were open source. A further review (Bataineh et al. 2018), aiming to determine the best CAD tool using a multi-criteria decision making process, considered ease of use, customer satisfaction, cost (initial and ongoing license), system requirements (software and hardware) and features.

A technical standards-oriented approach has been used (Eldrandaly 2007) in efforts to select the best GIS software according to several criteria derived from ISO/IEC 9126 and industry discussion. These were cost (upfront and perpetual), functionality, usability (split into understandability, learnability, and operability), reliability, and vendor. The range of useful functionality for GIS software identified included data import/export, data entry and editing, map design and composition, geographic query and analysis, image processing, and application development languages - that is, a programming environment for customised access to software functions.

Since the publishing of the previous paper (Eldrandaly 2007), ISO/IEC 9126 has been superseded by the ISO 25000 series. There are several software evaluation metrics described in ISO 25022 and ISO 25023; those of which deemed relevant to this investigation are set out in Tables 1 and 2 respectively, with any subsequent references to them using their ID. However, these metrics are designed for evaluation of software for specific and tightly defined tasks, rather than this exploratory review of a field. For example, Ef-1-G has the measurement function $X=A/B$, where A is the number of unique tasks completed and B is the total number of unique tasks attempted. The resulting count would be a valid comparison between two tools doing the same thing, but could only be subjectively applied in this case. As such the measurement functions are not repeated in the table, with the aim being to capture the sense of what characteristics are relevant for consideration in software tool evaluation.

**Table 1:** ISO 25022 evaluation metrics (ISO 2016a)

Name	Description
Effectiveness	
Tasks completed (Ef-1-G)	The proportion of the tasks that are completed correctly without assistance
Objectives achieved (Ef-2-S)	The proportion of the objectives of the task that are achieved correctly without assistance
Efficiency	
Task time (Ey-1-G)	The time taken to successfully complete a task
Time efficiency (Ey-2-S)	The efficiency with which users achieve their objectives over time when using the system
Cost effectiveness (Ey-3-S)	The cost effectiveness of the user
Productive time ratio (Ey-4-S)	The proportion of the time that the user is performing productive actions
Unnecessary actions (Ey-5-S)	The proportion of the actions performed by the user that were not necessary to achieve the task
User experience	
User pleasure (SPl-1-G)	The extent to which the user obtains pleasure compared to the average for this type of system
Ergonomic	
Physical comfort (SCo-1-G)	The extent to which the user is comfortable compared to the average for this type of system

**Table 2:** ISO 25023 evaluation metrics (ISO 2016b)

Name (ID)	Description
Functional completeness	
Functional coverage (FCp-1-G)	What proportion of the specified functions has been implemented?
Interoperability	
Data formats exchangeability (CIn-1-G)	What proportion of the specified data formats is exchangeable with other software or systems?
External interface adequacy (CIn-3-S)	What proportion of the specified external interfaces (interfaces with other software and systems) is functional?
Appropriateness recognisability	
Description completeness (UAp-1-G)	What proportion of usage scenarios is described in the product description or user documents?
Demonstration coverage (UAp-2-S)	What proportion of tasks has demonstration features for users to recognise the appropriateness?
Learnability	
Self-explanatory user interface (ULe-4-S)	What proportion of information elements and steps presented to the user enable common tasks to be completed by a first time user without prior study or training or seeking external assistance?
Operability	
Functional customisability (UOp-3-S)	What proportion of functions and operational procedures can a user customise for his convenience?
User interface customisability (UOp-4-S)	What proportion of user interface elements can be customised in appearance?
Modifiability	
Modification efficiency (MMd-1-G)	How efficiently are the modification made compared to the expected time?

The various software reviews and technical standards discussed present a range of useful functionalities for urban planning tools to possess and relevant characteristics by which their quality may be evaluated. These will inform the methodology by which this research conducts its own assessment.

## Methodology

Literature Review I (Sect. 2) identified the technical knowledge gap between citizens and urban planners as a barrier to participation by the former in planning. This gap is exemplified in the software tools used to complete planning tasks, prompting this review to study them in detail. It aims to survey a broad range of tools to identify their useful functionality with the goal of augmenting the urban planning process in a way that empowers communities towards enacting informed decisions. Literature Review II (Sect. 3) then identified relevant metrics for software evaluation, which have informed a schema for elucidating the beneficial functionality of each tool. This focus on barriers created by software design principles assumes a community who are willing to engage with software solutions, but digitally excluded communities are considered as part of future work.

### Functionality selection

The schema proposed to enable comparison between the software tools considered is shown in Table 3, informed by the discussed literature and the ISO 25000 series. Tools are only evaluated on their functionality that is directly linked to the schema. Temporal measurements are also contained within the standards, but they are omitted here; due to each tool having a slightly different purpose, this comparison was considered unfair.

**Table 3:** Tool evaluation schema

Characteristic	Definition
Cost	Categorises access as subscription, single purchase, or open source (Palomino et al. 2017; Bataineh et al. 2018; Eldrandaly 2007) Ey-3-S (ISO 2016a)
UI design	Included function explanations and intuitive actions (Al-Douri 2018; ul Hussnain et al. 2020; O’Connell et al. 2023) SPl-1-G SCo-1-G (ISO 2016a) UAp-1-G UAp-2-S ULe-4-S (ISO 2016b)
Included data	Integrated datasets (ul Hussnain et al. 2020; O’Connell et al. 2023)
Data import/export	Number of supported interchange formats (El Meouche et al. 2013; ul Hussnain et al. 2020; Eldrandaly 2007) CIn-1-G (ISO 2016b)
Included calculations	Number, scope, and complexity of analyses (Behmanesh and Brown 2019; O’Connell et al. 2023; Eldrandaly 2007)
Automation features	Tasks completed with reduced or no user input (excluding calculations) Ey-1-G Ey-2-S Ey-3-S Ey-4-S (ISO 2016a)
Extensions/plugins	Number of other tools to which this tool connects (Palomino et al. 2017) CIn-3-S (ISO 2016b)
Customisation options	User-programmable functional or UI elements (Eldrandaly 2007) UOp-3-S UOp-4-S MMd-1-G (ISO 2016b)
Distinct features	Notable attributes of this tool specifically Ef-1-G Ef-2-S (ISO 2016a) FCp-1-G (ISO 2016b)
Limitations	Any significant omissions from the tool’s capabilities Ef-1-G Ef-2-S Ey-5-S (ISO 2016a) FCp-1-G (ISO 2016b)

Each metric in the schema warrants discussion as to how it promotes community-driven decision making. Regarding Cost, cheaper is evidently better as this reduces investment required to start using any tool, which can be a significant barrier to entry if many copies of a tool are required or if a community does not have access to much funding. Subscriptions may reduce initial costs and include ongoing technical support, but will add up over time, so acknowledging payment structure is also relevant.

The User Interface (UI) Design is the next potential barrier to entry. If a first-time user is presented with a confusing layout of buttons with no explanation as to how it is intended that they accomplish even the most basic of functions, their engagement with the tool is likely to be short. While all software will possess a learning curve, efforts to keep it as shallow as possible are noted.

The provision of Included Data within a tool removes a task the user would otherwise have to complete themselves. In the case of users without much technical knowledge, this is also likely to include data they were unaware of, such as local regulations. Seamless integration of this data into a tool can be an easy way to educate communities and ameliorate their not knowing what they don’t know.

There are myriad file formats and data types used in urban planning practice. Data Import/Export between tools has thus become necessary for workflows to be practical, particularly for non-technical users who are less familiar with (and less interested in understanding) the intricacies of data interoperability.

Included Calculations and Automation Features provide similar boons in that they enable a tool to accomplish a task without user effort. There is potential for these tasks to include those which form part of planners’ technical education, removing the need for the public to receive the same education and thereby reducing the time it takes to get communities engaged with these tools.

Extensions/Plugins can streamline the design process by providing direct access from one tool to several others. This ease of use is helpful for everybody, but those less familiar with working across the multiple industry software packages will find it particularly advantageous.

The utilisation of these tools by communities is likely to be a new use case for many of them. Customisation Options provide functionality for adding the small changes identified on a case by case basis that soften the transition to their new application.

Where a tool does something special, this is noted among the Distinct Features. By definition this will be different in every case, but there will be a particular focus on features that grant capability to non-technical users.

The Limitations of a tool are important to note, as this may highlight additional barriers to entry that have workarounds in their professional use, but that become sizeable obstacles when introduced to communities. They are also likely to be considered as possible end users while the tools are being designed, so there may be limiting aspects specific to them that necessitate acknowledgement.

### Tool selection

The selection of software tools for this review is shown in Tables 4 and 5, with a split between established and emerging urban planning tools. The established tools are those explicitly built for and well utilised in the urban planning industry, covering GIS software and Rapid Concept Generation and Analysis (RCGA) tools; these are the types of tools discussed in the literature relating to software evaluation (El Meouche et al. 2013; Behmanesh and Brown 2019). The emerging tools, with functionality potentially beneficial to the industry but not yet experiencing commonplace use within it, are split into interoperability platforms and videogame engines; the need for the former has become evident as more industry software is created, the latter is beginning to see discussion in public participation literature (Cooney and Raghavan 2022).

The selection of tools for review within each category aims to capture the functionality on offer in that sector, and so begins with the most popular tools followed by alternatives with relevant innovations. Some further tools are acknowledged without detailed analysis, either due to having effectively identical functionality to another tool or limited relevance to this review, though they may be of interest to some readers.

**Table 4:** Established tools

Category	Tools
GIS software	ArcGIS, Google Earth, MapBox, QGIS, GRASS GIS, Google Earth Engine, Scribble Maps
Rapid concept generation and analysis	Autodesk Forma, ArcGIS CityEngine, ArcGIS Urban, Giraffe Urban Analytics, Blocktype, Hektar, TestFit, VU.CITY, PLACEMAKE.IO

**Table 5:** Emerging tools

Category	Tools
Interoperability platforms	Speckle, NVIDIA Omniverse
Videogame engines	Unreal Engine 5, Cities Skylines II

Each category is summarised with a comparison table, where every tool is given a $\checkmark$ or X depending on whether it makes a contribution to each characteristic of the schema (except cost, which is simply stated). If that contribution is particularly significant a $\bigstar$ is noted. For limitations, the $\checkmark$ means that limitations exist; $\bigstar$ denotes a significant limitation.

## GIS software

The tools within this category serve a range of different purposes, but they are typified by their ability to work with geospatial data, generally enabling its “capture, modeling, storage, retrieval, sharing, manipulation, analysis, and presentation” (Eldrandaly 2007), with the last of these often conducted through locating the data and subsequent analysis on a map. This range of purposes is too broad to exhaustively analyse here, but the functionality communities would experience in their use of the tools is examined in detail with regard to the schema. The tools most prevalent in industry (G2 2024) are analysed first, followed by alternative innovative approaches.

### ArcGIS (Esri Inc 2024a)

**Fig. 2:**
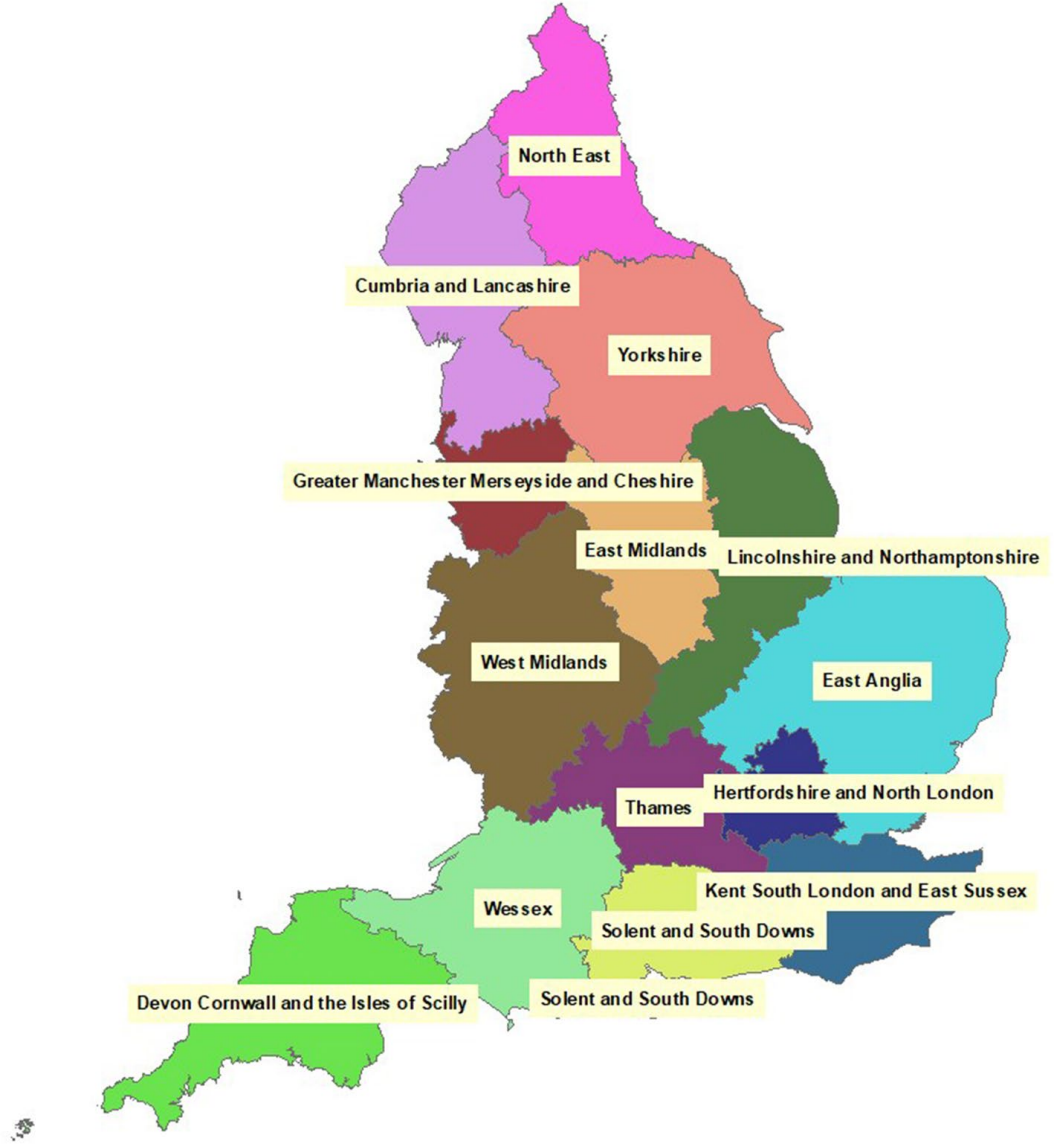
A simple map in ArcGIS, using an included dataset of administrative boundaries in England

The highest-user satisfaction tool according to one survey (G2 2024), ArcGIS is one of the most ubiquitous GIS platforms in industry. An example of its basic use is shown in Fig 2, with its functionality evaluated against the schema metrics in Table 6.

**Table 6:** ArcGIS

Cost	Range of annual subscriptions depending on required functionality for each user; £170 for the ability to view (but not edit) projects, £6000 for full feature access (Esri Inc 2024f)
UI design	Typical GUI, with functions accessible via customisable toolbars
Included data	ArcGIS online has some datasets, including OpenStreetMap
Data import/export	Data Interoperability extension supports over 110 spatial data formats, including GML, XML, WFS, and DWG/DXF (Esri Inc 2024d)
Included calculations	Various geospatial processing algorithms
Automation features	Python scripting or ModelBuilder functionality for automated geospatial processing workflows
Extensions/plugins	Developer API (Esri Inc 2024e)
Customisation options	Custom apps can be developed with App Builders to enhance interaction with GIS data (Esri Inc 2024i)
Distinct features	The variable pricing structure may provide efficient community access if the method of decision making utilises superusers, however, it could also be abused to suffocate community agency
Limitations	A steep learning curve typical of software designed for professionals

### Google Earth (Google LLC 2024d)

**Fig. 3:**
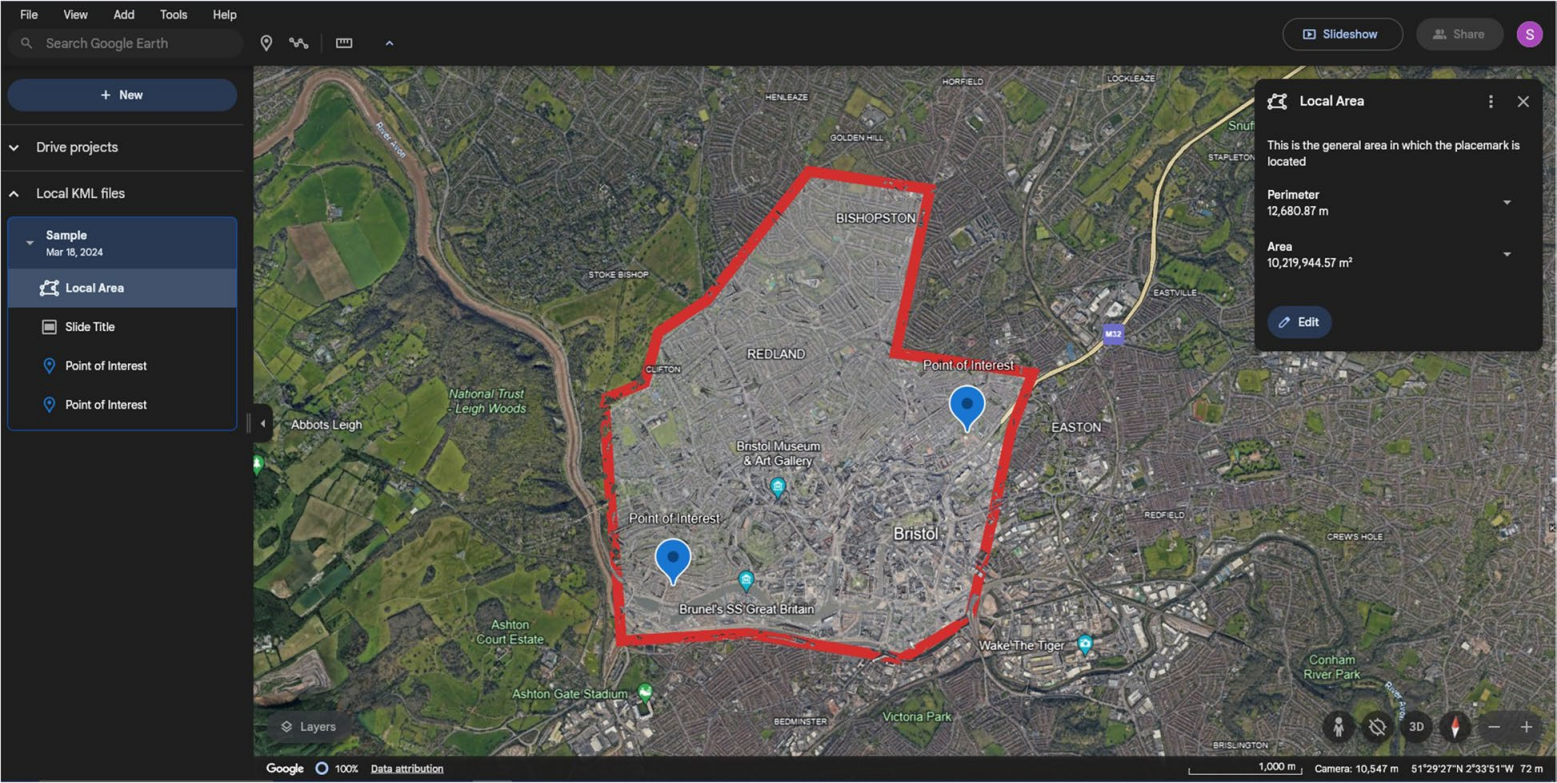
A sample Google Earth project, showing a travel area and two points of interest within it

A widely popular GIS tool (G2 2024), this is a simple web application for marking locations of interest and creating derivative presentations, intended to be used for holiday planning. The interface is shown in a sample project in Fig. 3 and its evaluation against the schema is given in Table 7.

**Table 7:** Google Earth

Cost	Free
UI Design	Simple web or desktop application displaying an interactive globe (Google LLC 2023)
Included data	Google satellite map data, Google Maps location data
Data import/export	User images can be imported for presentation slides KML (geographic variant of xml) file export
Included calculations	Drawn polygon area and perimeter, distance measurement
Automation features	None
Extensions/plugins	None
Customisation options	None
Distinct features	The user experience is intended to be accessible to any user, not just professionals
Limitations	A reduced amount of functionality, but the application is intentionally simple

### MapBox (MapBox 2024b)

MapBox is a set of APIs and SDKs for location intelligence, with services focused around navigation (MapBox 2024f), maps (MapBox 2024e), search (MapBox 2024h), and data provision; these enable developers to add geospatial data to their applications. It is evaluated in Table 8.

**Table 8:** MapBox

Cost	Starts free, with costs scaling by usage (MapBox 2024d)
UI design	As a set of APIs and SDKs, there is no UI; the end user experience depends on what applications are built with the services
Included data	Traffic (MapBox 2024i), population movement (MapBox 2024g), geospatial boundaries (MapBox 2024a)
Data import/export	GeoTIFF import for map tile data Each API functions by exporting its respective data
Included calculations	Navigation (including route finding, travel times, and logistics optimisation)
Automation features	API calls can be automated
Extensions/plugins	As an API service, MapBox effectively is a plugin for any internet-connected tool
Customisation options	None explicitly, but the APIs will be integrated into applications designed entirely by developers which are thus fully customised
Distinct features	MapGPT, an AI navigation assistant that can interact with locations along a route and with the vehicle (MapBox 2024c)
Limitations	Requiring web developer-level knowledge to access, this is not a standalone tool that can be used by the general public

### QGIS (QGIS 2024b)

**Fig. 4:**
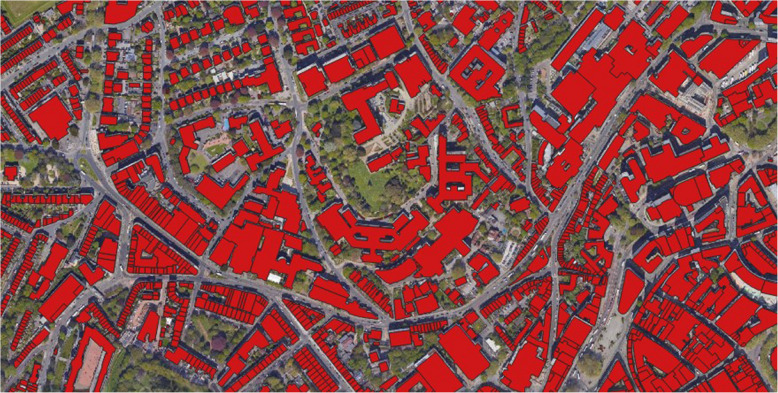
A map made in QGIS, with building polygons from OpenStreetMap over a Google Satellite image

A typical QGIS workflow consists of geospatial data import followed by creation of a map displaying that data, an example of which is shown in Fig. 4, with calculations and further analysis also optionally included. QGIS is evaluated in Table 9.

**Table 9:** QGIS

Cost	Free and open source (QGIS 2024a)
UI design	Typical GUI with functions accessible via customisable toolbars
Included data	None
Data import/export	Vector and raster geospatial data, geospatial database, geopackage, and point cloud import Image and pdf export
Included calculations	Several more detailed analyses are built-in
Automation features	None
Extensions/plugins	OpenStreetMap and Google Earth for geospatial data import, GRASS GIS for further calculations, Speckle for interoperability
Customisation options	Users can write their own plugins and can define custom forms and actions for input
Distinct features	There are myriad user-developed plugins available due the open source nature of the tool (Pasotti and Kartoza 2024)
Limitations	A steep learning curve typical of software designed for professionals

### GRASS GIS (GRASS Development Team 2023)

While broadly similar to QGIS, GRASS GIS has a particular focus on analytical calculations rather than pure cartography; its evaluation is presented in Table 10.

**Table 10:** GRASS GIS

Cost	Free and open source
UI design	The typical experience of toolbar icons for accessing functions A command line interface is provided in a separate window, which launches on startup and within the main program window, so every function can thus be accessed via a toolbar icon or a corresponding command
Included data	Sample datasets
Data import/export	Raster or vector data import and export
Included calculations	Assorted geospatial analyses
Automation features	Python API which can run any of the tool’s functions
Extensions/plugins	Assorted user-created addons (GRASS Development Team 2024)
Customisation options	As an open source tool, the user can develop any addition
Distinct features	Depth of geospatial analyses
Limitations	A steep learning curve typical of software designed for professionals

### Google Earth Engine (Google LLC 2024e)

Google Earth Engine is similar to GRASS GIS, with its online UI based entirely around programming and running analysis scripts. This functionality is evaluated in Table 11.

**Table 11:** Google Earth Engine

Cost	Free for non-commercial use, monthly subscriptions for professional users start at $500 (Google LLC 2024b)
UI design	A web-based interface containing a map viewer and JavaScript code editor environment (Google LLC 2024f), as well as a python library, REST API, and command line tool which can each be used as the interface
Included data	Google Earth Engine Data Catalog, publicly containing over 90 petabytes of satellite images and more than a thousand geospatial datasets (Google LLC 2024c)
Data import/export	GeoTIFF or TFRecord import for raster data, shapefile or csv for tabular data, with the same data types available for export
Included calculations	Various geospatial calculations are included in the programming libraries, which can be run on Google’s cloud servers (Google LLC 2024f)
Automation features	As a programmable tool, the user can execute batch scripts
Extensions/plugins	There is a QGIS integration for the Python API, and several other partners are listed (Google LLC 2024b)
Customisation options	The user can create their own scripts for running analyses and apps for viewing their results
Distinct features	The range of supported programming languages
Limitations	Earth Engine is an online only tool and requires programming knowledge to utilise

### Scribble Maps (Scribble Maps 2024c)

An online tool for quickly annotating and sharing maps, enabling geospatial data visualisation, Scribble Maps is evaluated against the schema metrics in Table 12.

**Table 12:** Scribble Maps

Cost	Limited features available free, annual subscriptions for further functionality start at $168 (Scribble Maps 2024b)
UI design	Web based with functions accessed via toolbars
Included data	Selection of maps including Google Maps and OpenStreetMap, USA parcel data available with paid subscriptions
Data import/export	Image, shapefile, KML, GeoJSON, and spreadsheet/csv (coordinates) import Email and web embedding for map export
Included calculations	Route optimisation, some simple geometric calculations
Automation features	None
Extensions/plugins	Connects to Google Sheets, Zapier, Salesforce, BigQuery, and Hubspot for data import
Customisation options	Users can write their own plugins to customise the UI (Scribble Maps 2024a)
Distinct features	Drawing functions for map annotation are very easy to use
Limitations	Lack of detailed calculation capabilities and automation features

### Others

BatchGeo (BatchGeo LLC 2024) is another popular tool that adds a few data processing functions to Google Maps. Oracle Spatial (Oracle 2024) is a geospatial database, through which data can be visualised with some geometric calculations possible. Maxar (Maxar Technologies 2024) and Cesium (Cesium GS Inc 2024) provide high-fidelity satellite and 3D data respectively, but as they provide data without any additional processing they are not considered for further analysis here.

### Discussion: the original geospatial approach

The comparison of the GIS tools is given in Table 13, with the results effectively splitting them into two groups. Google Earth and Scribble Maps provide fewer features in a simple UI, whereas the others are more complex but have a steep learning curve as a result (this is the recurring significant limitation). This explains their respective use cases: the more technical tools are used almost exclusively by professionals, with the other two actively marketed for all users. These results thus exemplify the current limitations on public participation identified in the literature and underline the need to enable communities access to more powerful computational tools.

**Table 13:** Summary comparison of GIS software functionality

Tool	Cost	UI design	Included data	Data import/export	Included calculations	Automation features	Extensions/plugins	Customisation options	Distinct features	Limitations
ArcGIS	£170 to £6000/yr	$$\checkmark$$	$$\checkmark$$	$$\checkmark$$	$$\checkmark$$	$$\checkmark$$	$$\checkmark$$	$$\checkmark$$	$$\bigstar$$	$$\bigstar$$
Google earth	Free	$$\bigstar$$	$$\checkmark$$	$$\checkmark$$	$$\checkmark$$	X	X	X	$$\checkmark$$	$$\checkmark$$
MapBox	Starts free	X	$$\bigstar$$	$$\checkmark$$	$$\checkmark$$	$$\checkmark$$	$$\bigstar$$	$$\checkmark$$	$$\bigstar$$	$$\bigstar$$
QGIS	Free	$$\checkmark$$	$$\checkmark$$	$$\checkmark$$	$$\checkmark$$	$$\checkmark$$	$$\bigstar$$	$$\checkmark$$	$$\checkmark$$	$$\bigstar$$
GRASS GIS	Free	$$\checkmark$$	$$\checkmark$$	$$\checkmark$$	$$\bigstar$$	$$\checkmark$$	$$\checkmark$$	$$\checkmark$$	$$\checkmark$$	$$\bigstar$$
Google earth engine	$500/mth	$$\checkmark$$	$$\checkmark$$	$$\checkmark$$	$$\checkmark$$	$$\checkmark$$	$$\checkmark$$	$$\checkmark$$	$$\checkmark$$	$$\bigstar$$
Scribble maps	Starts at $168/yr	$$\bigstar$$	$$\checkmark$$	$$\checkmark$$	$$\checkmark$$	$$\checkmark$$	$$\checkmark$$	$$\checkmark$$	$$\checkmark$$	$$\checkmark$$

## Rapid concept generation and analysis

Increased ease of use for software does not only benefit non-technical users. At the initial feasibility and concept generation stages of the design process, speed is often more productive than in-depth computational analyses. This has led to creation of a range of tools for rapid decision making, particularly in the real estate sector where the goal is understanding the value of land and of any future developments upon it. Each tool facilitates site selection, quick placement of simple buildings, and some subsequent calculations. It is hypothesised that this methodology has benefit for informing community decision making, and so a range of approaches are compared below.

### Autodesk Forma (Autodesk Inc 2024b)

**Fig. 5:**
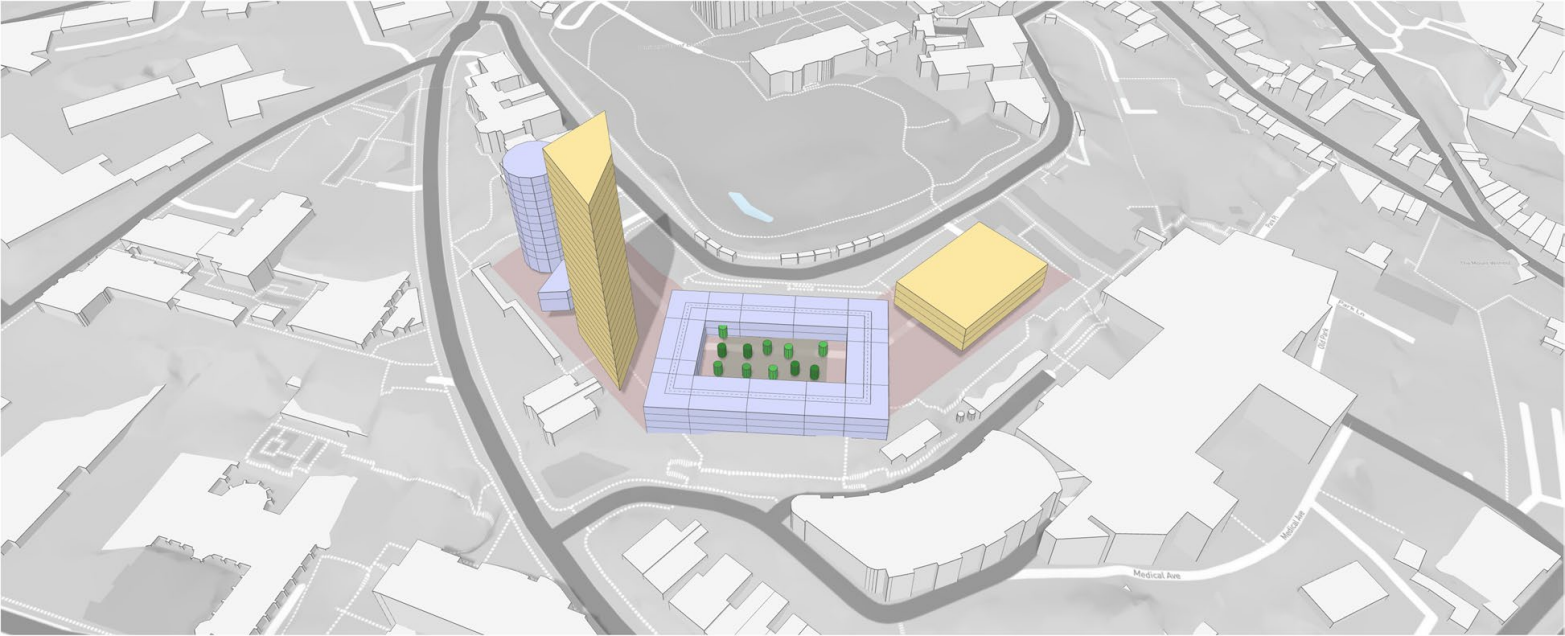
A sample model in Autodesk Forma

Autodesk’s offering in this space is a web browser-based tool in which the user selects a site and adds a model of their proposed development containing buildings, vegetation, and other areas, as shown in Fig. 5, on which calculations are then conducted. Its evaluation against the schema is shown in Table 14.

**Table 14:** Autodesk Forma

Cost	Subscription at £192 monthly, £1518 yearly, or £4554 to lock in a three-year price (Autodesk Inc 2024a)
UI design	Site boundary and other objects can be drawn quickly with a few clicks
Included data	An OpenStreetMap connection for contextual topological and 3D building data
Data import/export	User data can be added from.obj, IFC,.dxf,.jpg, and.png files
Included calculations	Site area, sun hours, daylight potential, wind, microclimate, operational energy, and noise - with examples in Fig. 6 - however, only the results are provided; except in the case of energy efficiency, the background model used to reach them is inaccessible
Automation features	None
Extensions/plugins	Revit, Dynamo, and Rhino
Customisation options	Users can develop their own plugins
Distinct features	The built-in calculations run rapidly
Limitations	Does not offer much functionality compared to other tools

**Fig. 6:**
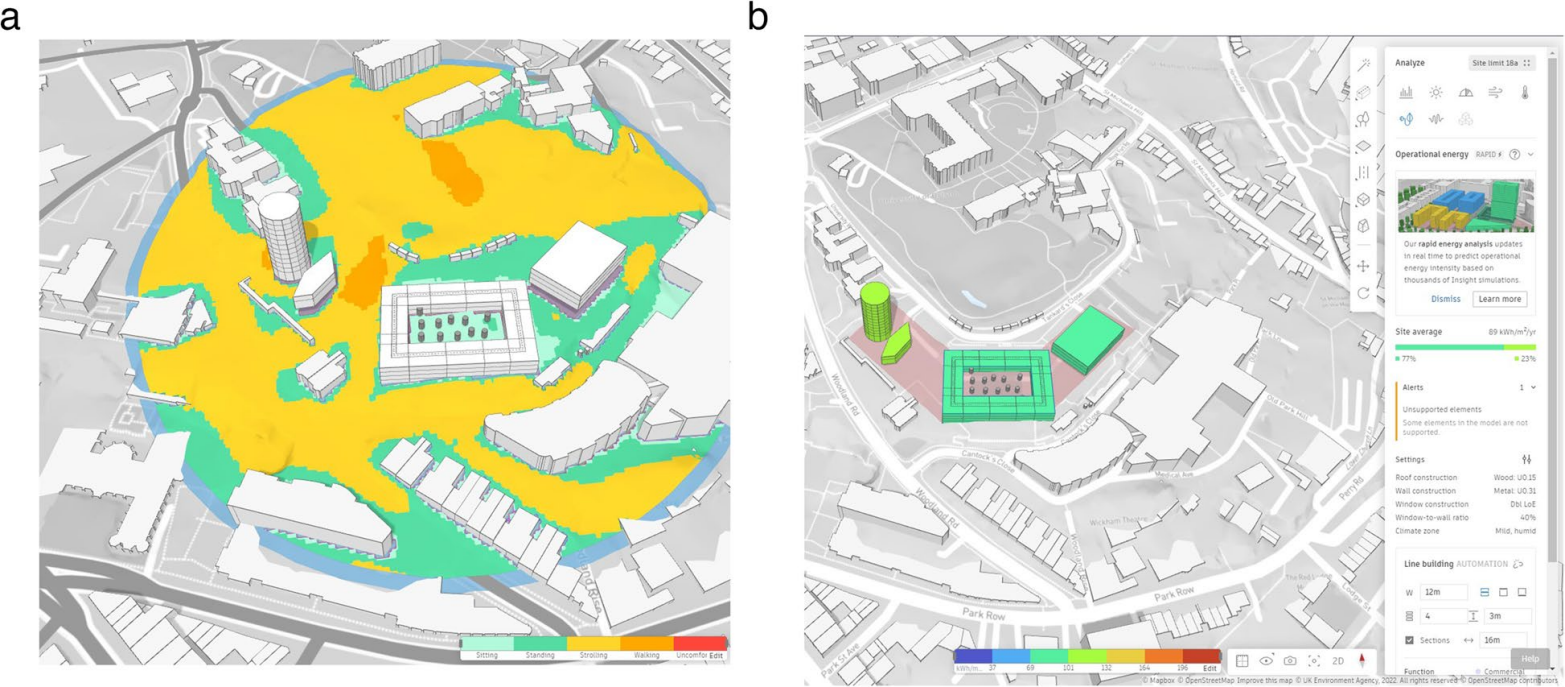
Example analyses for a site in Autodesk Forma

### ArcGIS CityEngine (Esri Inc 2024b)

Built on ArcGIS, CityEngine focusses on parametric modelling at the city scale for design visualisation through CGA, its bespoke visual scripting language. It is evaluated in Table 15.

**Table 15:** ArcGIS CityEngine

Cost	Annual subscription of £3591 (Esri Inc 2024c)
UI design	Typical GUI, with functions accessible via customisable toolbars
Included data	None
Data import/export	ArcGIS and OpenStreetMap data import, and supports various CAD files Models can be exported to the web, VR, game engines, or a geospatial database, and CGA scripts can be saved and shared
Included calculations	Basic geometric analyses, zoning visualisation
Automation features	Procedural city generation with CGA
Extensions/plugins	Connects to ArcGIS Online and ArcGIS Urban
Customisation options	None
Distinct features	CGA scripting language for parametric, procedural city generation
Limitations	Lack of detailed analysis tools

### ArcGIS Urban (Esri Inc 2024j)

Another ArcGIS tool, this conducts 3D modelling of cities with functionality that is less powerful than CityEngine, but with greater capability for urban analytics calculations. The two should therefore be used in conjunction for maximum productivity. It is evaluated in Table 16.

**Table 16:** ArcGIS Urban

Cost	Undisclosed, but AcrGIS CityEngine is included (Esri Inc 2024g)
UI design	An online only tool, with a simple interface
Included data	Example cities with detailed zoning, land use, and building types, which can be duplicated and customised, as well as Living Atlas (ArcGIS Living Atlas of the World 2024) indicators, such as diversity, education, and housing density, for USA-based projects
Data import/export	Zoning, land use, and building types data import from ArcGIS Online Project export to ArcGIS StoryMaps, a tool for presenting geospatial information
Included calculations	Zoning and land use visualisation, a range of analytics for measuring development impact such as population, jobs, and revenue, and shadow and line-of-sight calculations
Automation features	ArcGIS Urban API allows automation of repetitive tasks
Extensions/plugins	Connects to ArcGIS tools, particularly CityEngine
Customisation options	ArcGIS Urban API enable development of custom functionality and new tool integrations
Distinct features	Citizen participation functions enabling communities to view proposals in 3D and give feedback through annotations and questionnaires (Esri Inc 2024h)
Limitations	Effectively requires commitment to the ArcGIS ecosystem

### Giraffe Urban Analytics (Giraffe Technology Pty Ltd 2024a)

**Fig. 7:**
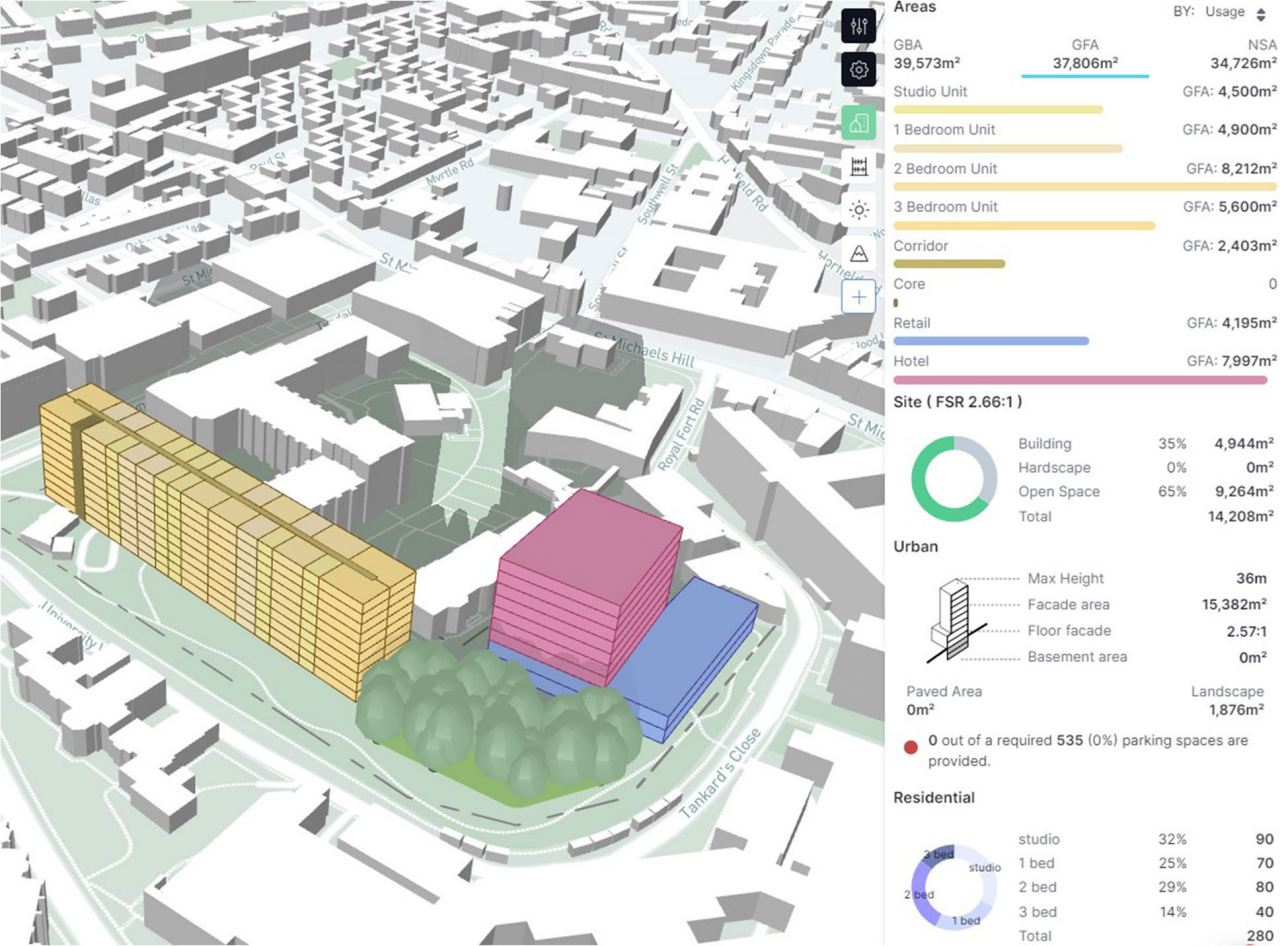
Area calculations for an example site in Giraffe

Broadly similar in use to Autodesk Forma, Giraffe comes with more features regarding data import, drawing objects, and customisation. A sample project is shown in Fig. 7, with its evaluation in Table 17.

**Table 17:** Giraffe Urban Analytics

Cost	Limited features available free, annual subscriptions for further functionality start at $3000 (Giraffe Technology Pty Ltd 2024b)
UI design	Buildings are quick to draw and edit with an intuitive interface and controls
Included data	Contextual geospatial data from OpenStreetMap, traffic data from MapBox, and utility locations and zoning areas from local authorities where they have provided it
Data import/export	Users can upload their own GIS data
Included calculations	Area calculations, shown in Fig. 7, demographics, solar analysis, rent feasibility, and sale feasibility (the last two requiring extra user data)
Automation features	The calculation system is customisable, which can automate some tasks if the user designs it to
Extensions/plugins	Speckle
Customisation options	Users can extend the calculation system through adding operations on the data available in the model; they can also develop their own extensions for the tool
Distinct features	Flow, a parametric geometry system allows definition of dynamic building layouts, of which the yellow building in Fig. 7 is an example; it is an apartment block with a mixture of dwelling units Usages, a system which allows a class-based definition of various building parameters and assumptions, with built-in and user-defined options that inform the calculations
Limitations	The combination of high cost and highly restricted free tier

### Blocktype (Blocktype 2024a)

**Fig. 8:**
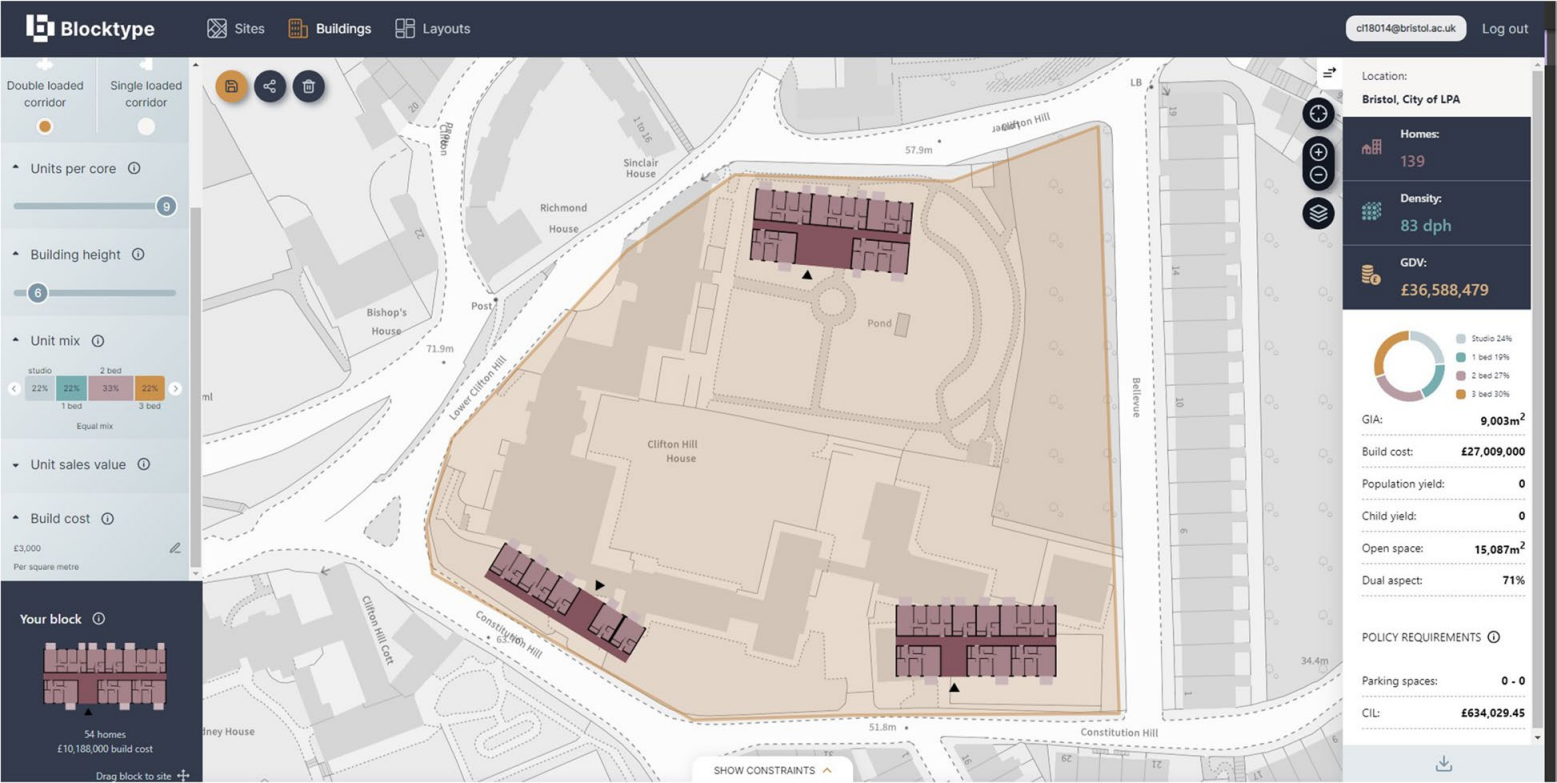
Example use of Blocktype

Taking a slightly different approach, Blocktype forgoes 3D visualisation and detailed customisation in favour of delivering a rapid process for ascertaining a site’s gross development value. The choice of buildings is limited to a selection of preset floor layouts which the user can place on their site, as shown in Fig. 8. This functionality is evaluated in Table 18.

**Table 18:** Blocktype

Cost	Annual subscription of £1068 (Blocktype 2024b)
UI design	The tool is 2D only, but everything is very simple and streamlined
Included data	OS maps or Google Satellite for 2D site data, property boundaries, conservation areas, listed buildings, and flood zones
Data import/export	None
Included calculations	Gross development value, number and density of homes, community infrastructure levy, area, build cost, and child yield, with no methodology for reaching these numbers given, and they run on user-supplied assumptions
Automation features	None
Extensions/plugins	None
Customisation options	None, although the developers have indicated openness to working with users to add their specific use cases
Distinct features	This tool is designed to be (and is) very quick to use
Limitations	Reduced features in favour of high speed use

### Hektar (Parametric Solutions AB 2024a)

Hektar is a generative design tool aimed at the early stage of city-scale concept generation. When provided with a site boundary and some simple building constraints, an algorithm creates several potential site layouts, which can then be compared by the user. It is evaluated in Table 19.

### TestFit (TestFit Inc 2024b)

**Fig. 9:**
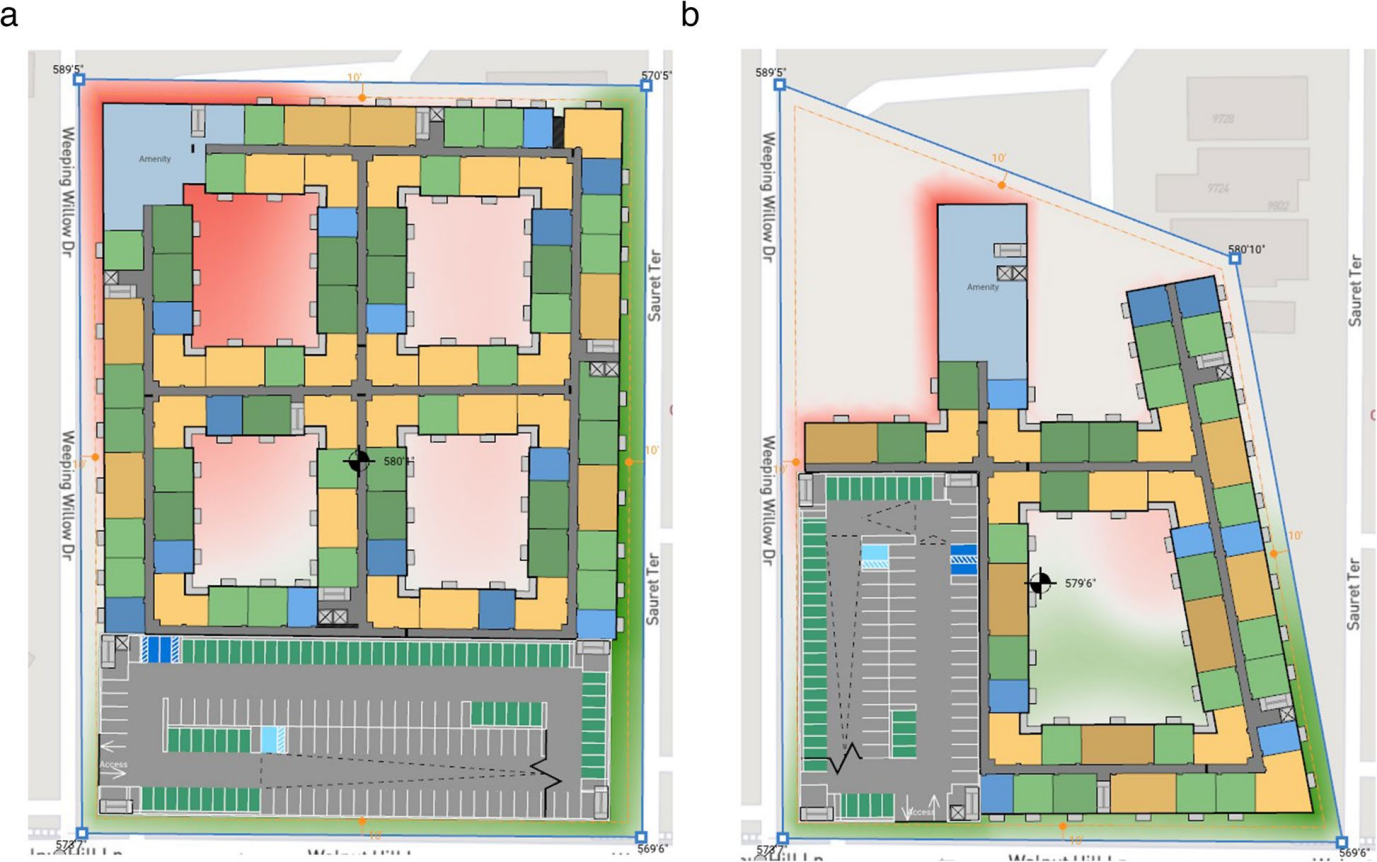
TestFit’s dynamic response to an adjustment of a site’s top right corner

TestFit focusses on generating high-fidelity parametric proposals and provides basic analytics for site feasibility studies. An example of its dynamic response to design changes is shown in Fig. 9, with its evaluation against the schema given in Table 20.

**Table 19:** Hektar

Cost	Limited features available free, monthly subscriptions for further functionality start at €179 (Parametric Solutions AB 2024b)
UI design	An online tool with a simple interface
Included data	MapBox and OpenStreetMap for base map
Data import/export	Shapefile and.dxf file import for site boundary definition.obj,.3dm, and.csv design proposal export
Included calculations	Generative design of site layouts given constraints on site boundary, access points, preferred building type and size, and parking requirements Basic geometric calculations for each generated concept
Automation features	The aim of the tool is to automate the early concept design process
Extensions/plugins	None
Customisation options	None
Distinct features	Generative design algorithm allows comparison of many design concepts
Limitations	This is a very new tool, with the product not currently widely accessible

**Table 20:** TestFit

Cost	Undisclosed; a free trial is available with limited features
UI design	Standalone software, but with a similarly intuitive UI to many of the web-based tools
Included data	MapBox base maps, USA land ownership parcels, elevation data, Zoneomics zoning data (Zoneomics 2023)
Data import/export	Image import for site boundaries.dxf,.skp,.glTF,.csv,.pdf for file export
Included calculations	Potential site layouts given a boundary and preferred unit mix Basic geometric and real estate focused analytics
Automation features	Analytics calculations and building layouts readjust dynamically according to user edits
Extensions/plugins	Revit and Enscape (TestFit Inc 2024a) add-ins
Customisation options	None
Distinct features	Dynamic response of generative algorithms when customising site proposals, including road layouts
Limitations	Limited customisability for more detailed analysis

### VU.CITY (VU.CITY limited 2024)

VU.CITY provides 3D models of 25 UK and international cities into which users can place their models and visualise them through various lenses. Its evaluation is given in Table 21.

**Table 21:** VU.CITY

Cost	Undisclosed
UI design	Access to this tool proved prohibitively expensive, preventing this being fairly assessed
Included data	3D models of 25 UK and international cities accurate up to 15 cm of detail Planning-related data layers such as listed buildings and conservation areas
Data import/export	User model import from Revit, Rhino, SketchUp, and 3DS Max
Included calculations	Sunlight and microclimate analyses are included, along with visualisations for protected views and zones of theoretical visibility (where a proposed development will be visible from)
Automation features	SiteSolve, an additional feature developed in partnership with Ramboll, which automatically suggests design options for a site and set of given criteria
Extensions/plugins	None
Customisation options	None
Distinct features	The tool includes a development timeline for a site and its nearby developments, as well as collaboration and presentation functionality
Limitations	Only works in the cities for which models have been provided

### PLACEMAKE.IO (PLACEMAKE.IO Ltd 2024)

**Fig. 10:**
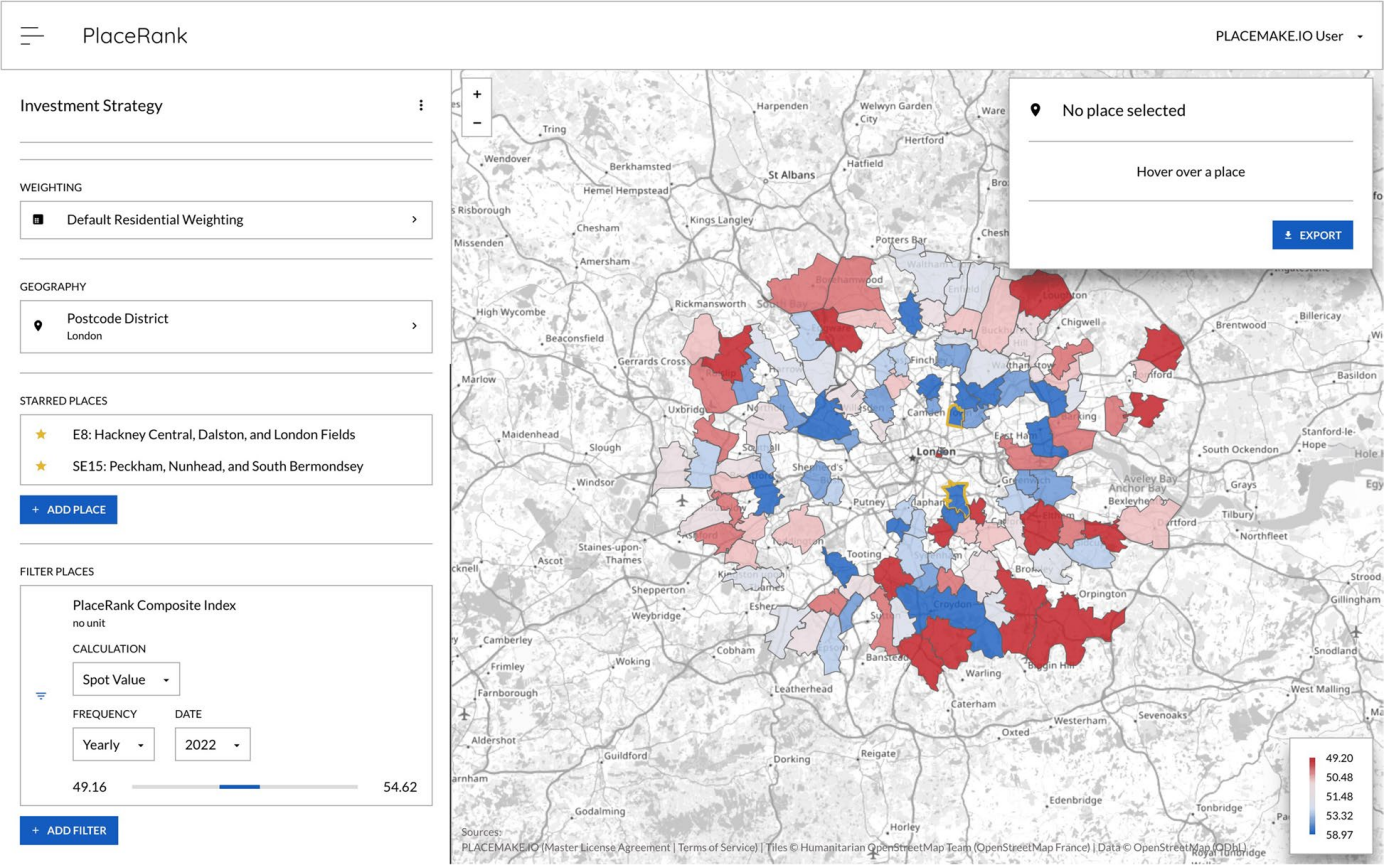
Example PlaceRank map (PLACEMAKE.IO Ltd 2023)

PLACEMAKE.IO focusses exclusively on data and analytics for assisting real estate investment decisions, such as dealmaking and portfolio management, through various models and indices assessing the current and future states of specific assets and broader markets. Users interact with this analysis through PlaceRank, a dashboard platform that provides access to the data and ranks locations based on user-defined criteria, or a custom integration into their own platform. A sample output is shown in Fig. 10; it is evaluated in Table 22.

**Table 22:** PLACEMAKE.IO

Cost	Undisclosed
UI design	User experience mostly consists of highly configurable dashboards
Included data	Everything deemed necessary to the decision making process, sourced from public datasets and leading market data providers
Data import/export	Users can share their data for integration into the data engine.csv and image (.png) data export
Included calculations	Some proprietary indices are provided which are derived from the included data, such as a walkability index, though no precise methodology for how these are calculated is given Users may also conduct simple analyses based on how they construct the various dashboards and maps
Automation features	None
Extensions/plugins	None
Customisation options	A connection from the data analysis to a user's private system can be constructed
Distinct features	All data gathering and compilation is succinctly provided in a single source of truth
Limitations	A proprietary, black box system reduces its inspectability

### Others

Modelur (Modelur 2024), CityCAD (CityCAD Technologies Limited 2024), and 3D Cityplanner (Strategis Groep bv 2023) are other 3D parametric building modellers with some basic further analysis but they are quite similar in both form and function to the tools already discussed.

Delve (Google LLC 2024a), by Google’s Sidewalk Labs, is to use AI to calculate potential site layouts in a method similar to VU.CITY’s SiteSolve or Hektar. However, it is currently only a webpage without any accessible version.

While there are also many tools which enable 3D modelling and visualisation, they have not been considered above unless they do something specific to urban planning; some relevant options are noted here. Rhino Grasshopper (Robert McNeel & Associates 2024) is the current industry standard for parametric modelling, which many of the discussed tools incorporate. Blender (Blender Foundation 2024), 3D modelling software that featuring rendering and animation, is open source with a large community developing extensions for it. Of particular note among these are BlenderBIM (Blender Community 2024), BlenderGIS (domlysz and Blender Community 2020), and ICity (procedural city generation) (ICity 2024), which together bring it close to software that would be considered in this review. However, its steep learning curve and the prevalence of more dedicated tools mean that it unlikely to be the best choice. Other 3D modelling tools include SketchUp (Trimble Inc 2024), Lumion (Lumion 2024), and TwinMotion (Epic Games Inc 2024a).

### Discussion: accessible design methodologies

The summary of the comparison is given in Table 23. Many of the particularly notable Distinct Features identified relate to parametric modelling and generative design, with both of these having significant potential to enable non-technical users to engage with the design process. Parametric modelling, where most dimensions and attributes of a design are defined relative to each other rather than as absolute values and where a few main values drive everything else, allows models to be quickly reconfigured without much user input. This has prevented professional users from getting stuck recalculating technical details every time they want to make a small change, and could additionally help public users as they are also unlikely to understand how to conduct these recalculations. Generative design, where an algorithm or AI model creates potential solutions by itself, can provide similar time-saving benefits. This technology is new so the current quality of its output is variable, but it is also rapidly developing and can be expected to soon incorporate much of the technical and regulatory knowledge which users without a formal planning education lack.

ArcGIS Urban is the only tool to have explicit citizen participation features. A process is provided for obtaining community feedback in which the use of 3D models will prove more engaging than typical 2D presentations. However, there is no inherent guarantee that this feedback will be acted upon; it does not provide communities with significant power. Further development is needed to involve them earlier on in the design process, rather than enquiring of their opinion after core decisions have been made.

**Table 23:** Summary comparison of RCGA application functionality

Tool	Cost	UI design	Included data	Data import/export	Included calculations	Automation features	Extensions/plugins	Customisation options	Distinct features	Limitations
Autodesk Forma	Starts at £1518/yr	$\checkmark$	$\checkmark$	$\checkmark$	$\checkmark$	$\checkmark$	$\checkmark$	$\checkmark$	$\checkmark$	$\checkmark$
ArcGIS CityEngine	£3591/yr	$\checkmark$	X	$\checkmark$	$\checkmark$	$\checkmark$	$\checkmark$	X	$\bigstar$	$\checkmark$
ArcGIS Urban	Undisclosed	$\checkmark$	$\checkmark$	$\checkmark$	$\checkmark$	$\checkmark$	$\checkmark$	$\checkmark$	$\bigstar$	$\checkmark$
Giraffe Urban Analytics	Starts free	$\checkmark$	$\checkmark$	$\checkmark$	$\bigstar$	$\checkmark$	$\checkmark$	$\checkmark$	$\bigstar$	$\checkmark$
Blocktype	£1068/yr	$\checkmark$	$\checkmark$	X	$\checkmark$	X	X	X	$\bigstar$	$\checkmark$
Hektar	Starts free	$\checkmark$	$\checkmark$	$\checkmark$	$\checkmark$	$\bigstar$	X	X	$\bigstar$	$\checkmark$
TestFit	Undisclosed	$\checkmark$	$\checkmark$	$\checkmark$	$\bigstar$	$\checkmark$	$\checkmark$	X	$\bigstar$	$\checkmark$
VU.CITY	Undisclosed	$\checkmark$	$\bigstar$	$\checkmark$	$\checkmark$	$\bigstar$	X	X	$\bigstar$	$\checkmark$
PLACEMAKE.IO	Undisclosed	$\bigstar$	$\checkmark$	$\checkmark$	$\checkmark$	X	X	X	$\checkmark$	$\checkmark$

## Interoperability platforms

An issue with the variety of urban planning software now available arises from the fact that many professional workflows include multiple tools as each is the solution of choice for one of its functions. This raises the question of how data is to be transferred between disparate software packages, with FEA, visualisation, and business statistics being additional needs accomplished by a further set of tools. Solutions for interoperability have recently been developed, which would enable the best parts of all software to be amalgamated into a public participation-oriented planning methodology that should reduce the confusion arising from the array of tools and datasets among an audience especially susceptible to this.

### Speckle (AEC Systems Ltd 2024)

Speckle provides a web server to which user data from various architecture, engineering, and construction (AEC) software can be uploaded, converted, and downloaded. To handle this conversion, Speckle provides a range of plugins for the tools which it supports. Its functionality is evaluated against the schema metrics in Table 24.

**Table 24:** Speckle

Cost	Free and open source, with enterprise options for server hosting and technical support at an undisclosed price (AEC Systems Ltd 2021a)
UI design	A website with an interactive project viewer as shown in Fig. 11, as well as in-app plugins which often accomplish data transfer in a single click
Included data	None
Data import/export	Files from a diverse range of AEC software
Included calculations	None
Automation features	Speckle Automate, a set of APIs and SDKs for interacting with the web server - although this is not currently fully released
Extensions/plugins	Data import/export plugins for Revit, Grasshopper, PowerBI, Rhino, SketchUp, Excel, Dynamo, QGIS, Unreal Engine, ArcGIS and more (AEC Systems Ltd 2021b)
Customisation options	This is an open source tool, so everything is fully customisable and extendable - which is part of its intended use
Distinct features	A Github-style version control for models
Limitations	Not all of one tool’s data is always compatible with another and so is lost in conversion, requiring an understanding of the data from the workflow designer

**Fig. 11:**
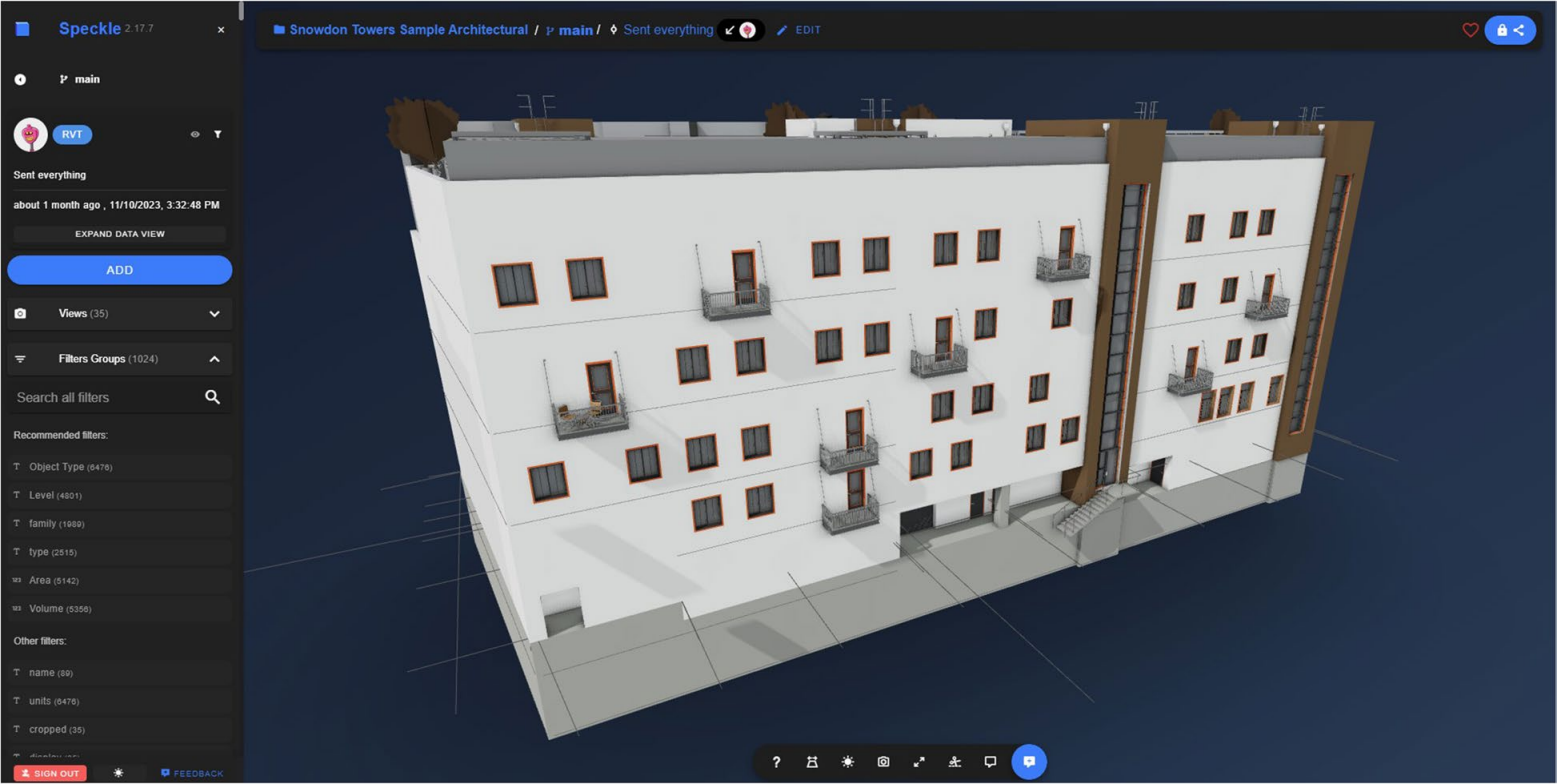
Default Revit model uploaded to Speckle’s web viewer

### NVIDIA Omniverse (NVIDIA Coporation 2024b)

A collection of APIs, SDKs, and proprietary NVIDIA software, Omniverse runs on Universal Scene Description (OpenUSD) and NVIDIA RTX. It focusses on 3D workflows, rather than explicitly AEC use cases, and thus interacts with a slightly different array of software, though digital twins are one of its target use cases. It is evaluated in Table 25.

**Table 25:** NVIDIA Omniverse

Cost	Includes a free tier; higher tiers with more features are available for an undisclosed price
UI design	The APIs and SDKs have no UI by nature, NVIDIA’s own applications provide a standard experience
Included data	None
Data import/export	Files from a wide range of 3D design software (NVIDIA Coporation 2024a)
Included calculations	None
Automation features	APIs can be used to automate repetitive tasks
Extensions/plugins	Data import/export plugins for Revit, ArcGIS CityEngine, SketchUp, Unreal Engine, and more (NVIDIA Coporation 2024a)
Customisation options	None explicitly, but the APIs can be integrated into apps designed entirely by developers which are thus fully customised
Distinct features	NVIDIA intend for Omniverse to be a sufficiently detailed simulation that robots can conduct reinforcement learning to accomplish real-world tasks (NVIDIA 2024); while this claim should be taken with a pinch of salt given current hype around AI, it is worth considering that it is made by NVIDIA
Limitations	Requires an RTX NVIDIA graphics card to run

### Others

Industry Foundation Classes (IFC) are a schema for data organisation; they are practically implemented as IFC files. This file format is designed to be universally intelligible to AEC software, enabling interoperability between them. However, the ubiquitous uptake required to make it effective did not occur, with the functionality provided by bespoke file formats proving more effective.

### Discussion: the need for communication

The comparison is given in Table 26, though there is nothing particularly surprising here. Speckle and Omniverse score well for data import/export and extensions/plugins, as this is their purpose; they do not include data or calculations as that is the purpose of the other tools to which they connect. The point to consider, then, is what value they could provide in being part of a larger urban planning workflow. While individual tools have some file interchange methods as detailed above, interoperability platforms present a complete approach to enabling an integrated workflow that streamlines the urban planning process for users without extensive experience in the full range of tools - with which the custom applications that can be built on top of Speckle could also aid.

**Table 26:** Summary comparison of interoperability platform functionality

Tool	Cost	UI design	Included data	Data import/export	Included calculations	Automation features	Extensions/plugins	Customisation options	Distinct features	Limitations
Speckle	Starts free	$\checkmark$	X	$\bigstar$	X	$\checkmark$	$\bigstar$	$\bigstar$	$\checkmark$	$\checkmark$
NVIDIA omniverse	Starts free	$\checkmark$	X	$\bigstar$	X	$\checkmark$	$\bigstar$	$\checkmark$	$\checkmark$	$\checkmark$

## Videogame engines

The use of videogame engines has begun to make its way into the AEC industry for visualisation of projects, providing a previously unattainable understanding of what they would look like when built and creating potential novel methods of communicating development proposals and the design thinking behind them to communities. The games themselves are also becoming increasingly realistic simulations while maintaining an experience intended to be enjoyable for non-technical players, warranting consideration of their use as urban planning tools.

### Unreal Engine 5 (Epic Games Inc 2024c)

**Fig. 12:**
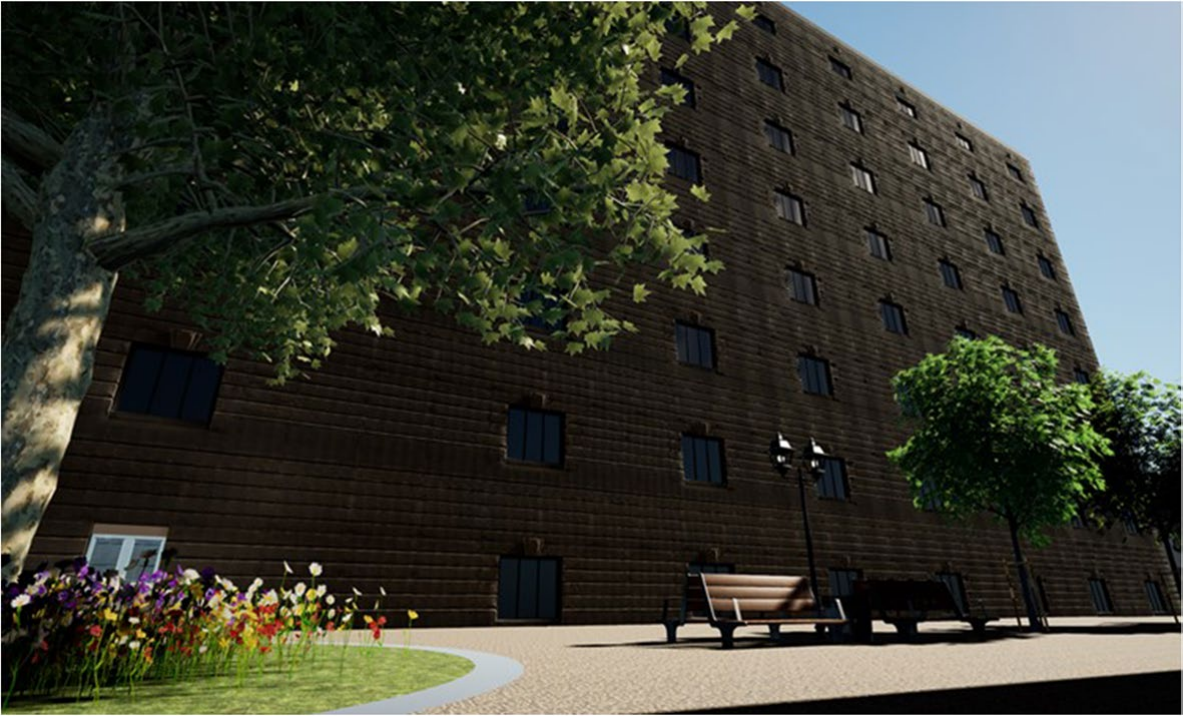
A mixed-use development modelled for virtual reality in UE5 (Hanan et al. 2023)

A video game development engine, Unreal Engine 5 (UE5) is targeted at the AEC industry as regards its visualisation capabilities and is now commonly used to produce graphics for stakeholder engagement, an example of which is shown in Fig. 12. While it does not explicitly provide any functionality intended for the design of cities, research has shown the processes involved in game development can be applied to the design of buildings (Hanan 2022); they could also be applied at the city scale. This functionality is evaluated in Table 27.

**Table 27:** Unreal Engine 5

Cost	Free for small projects, annual subscription of £1770 or a 5% royalty for businesses with over $ 1 m in revenue depending on their product (Epic Games Inc 2024d)
UI design	A typical experience with myriad functions accessible by toolbars and many keyboard shortcuts
Included data	None
Data import/export	Datasmith plugins for 3D data import, supporting Revit, SketchUp, IFC, Rhino and more (Epic Games Inc 2024b)
Included calculations	None
Automation features	Procedural generation functions for creating environments
Extensions/plugins	There is a storefront with a range of additional content One notable plugin is CityBLD (WorldBLD 2024), which utilises the procedural generation features to create detailed city environments and enables OpenStreetMap data import
Customisation options	As an open source tool, the user can create their own plugins
Distinct features	Unreal Engine provides an environment in which anything 3D can be created in precise detail
Limitations	There is nothing specific to urban planning included with the tool; it also has a very steep learning curve, but this is because it accomplishes myriad tasks

### Cities Skylines II (Paradox Interactive AB 2024c)

**Fig. 13:**
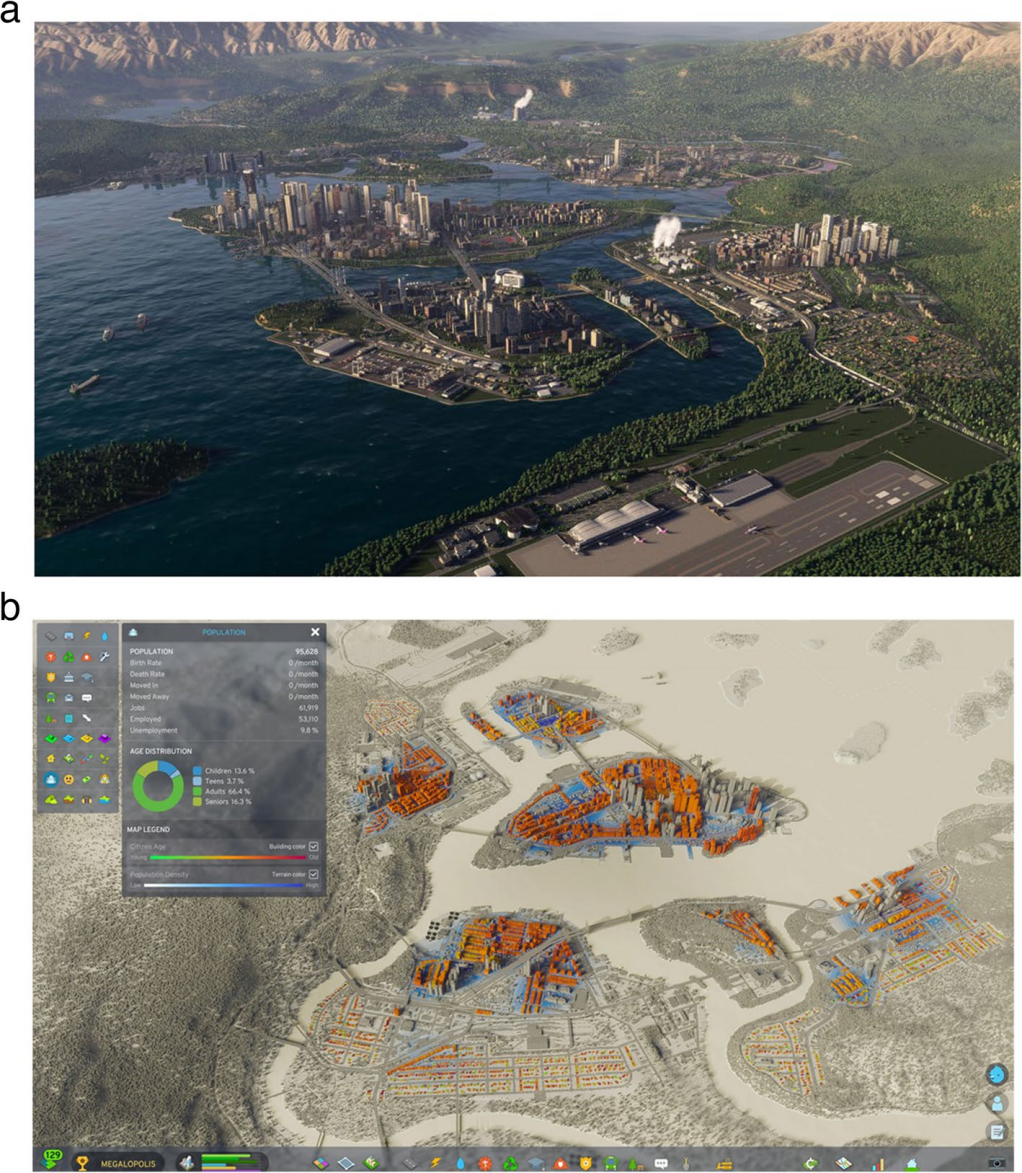
Gameplay screenshots from Cities Skylines II (Paradox Interactive AB 2024c)

A videogame where the player builds a virtual city, it includes simulation of a dynamic environment which aims to be realistic, as shown by the detailed model in Fig. 13a and corresponding data in Fig. 13b. The first edition of the game had a huge community creating additional content that could be imported into the game, and the second has a dedicated platform for this content, Paradox Mods (Paradox Interactive AB 2024b), through which the game’s underlying code can be modified to add further analyses in order to utilise the existing simulation engine to develop an accurate city analysis model. Its evaluation against the schema metrics is given in Table 28.

**Table 28:** Cities Skylines II

Cost	£41.99, or £74.99 for the ultimate edition (Paradox Interactive AB 2024a) Further content for the game is expected in future at an additional cost
UI design	As a video game, there is a focus on an intuitive UI usable by any player
Included data	Various simulation parameters as determined by the developers
Data import/export	Custom maps can have heightmaps imported as.png or.tiff files (Paradox Interactive AB 2024d) 3D content should soon be importable in various formats
Included calculations	Various city simulation calculations as determined by the developers
Automation features	Detailed city simulation
Extensions/plugins	Player-created content is available from Paradox Mods (Paradox Interactive AB 2024b)
Customisation options	Users can make their own content for the modding platform
Distinct features	The city simulation engine
Limitations	There will be deliberate inaccuracies in the simulation to provide a better gaming experience, and the game requires high-end hardware to run

### Others

Unity (Unity Technologies 2024) is another game development engine and the closest competitor to UE5. They are functionally equivalent for the purposes of this review, with UE5 discussed here due to author familiarity.

The SimCity videogame series (Electronic Arts Inc 2016) is the closest competitor to Cities Skylines II. However, it was not as popular and the last installment was released in 2013, as opposed to Cities Skylines II’s 2023 release.

### Discussion: a novel approach to citizen participation

The analysis of these tools is summarised in Table 29. UE5’s lack of dedicated urban planning functionality is a significant limitation, but the versatility of its design processes mean that it is a strong choice for implementing functionality not provided by any tool, should a community require something bespoke.

Cities Skylines II is potentially the most empowering tool for communities. It possesses a user interface designed for any level of knowledge that communicates complex information in a way that cannot be confusing or boring else player retention would be low. Its simulation engine automates much of the technical analysis inexperienced users would not understand and presents the higher-level decisions to them directly. While its accuracy and thus viability as a modelling tool needs further exploration before a final assessment of it can be made, any shortcomings identified can be rectified through use of the modding platform. This platform could also possibly be used to add further citizen participation features to create a tailored experience for communities.

**Table 29:** Summary comparison of videogame engine functionality

Tool	Cost	UI design	Included data	Data import/export	Included calculations	Automation features	Extensions/plugins	Customisation options	Distinct features	Limitations
Unreal Engine 5	Starts free	$\checkmark$	X	$\bigstar$	X	$\checkmark$	$\checkmark$	$\bigstar$	$\checkmark$	$\bigstar$
Cities Skylines II	Starts at £41.99	$\bigstar$	$\checkmark$	$\checkmark$	$\checkmark$	$\bigstar$	$\checkmark$	$\bigstar$	$\bigstar$	$\checkmark$

## Discussion

With the range of urban planning tools thus surveyed, how they might coalesce to improve citizen participation is now considered through identifying the most useful related functionality and elucidating the combination of tools that best delivers it.

The survey of GIS software confirmed the need identified in the literature for development of tools accessible to communities, finding that the current options were often technically challenging to use and thus only viable for professionals. However, this conflicted with the need for computational power to be retained, as the GIS software that was easy to use also accomplished less.

The RCGA applications provided two functionalities with potential to ameliorate this issue: parametric modelling and generative design. These can automate some of the technical analysis that inexperienced users would struggle to conduct, reducing both the time and effort required from them without limiting the depth of insight. These applications also generally provided more intuitive UIs when compared to the GIS software, such as Giraffe’s 3D modelling controls, PLACEMAKE.IO’s interactive dashboards, and ArcGIS Urban’s 3D presentations for citizen participation.

An aspect that needs further consideration is the emergent behaviour resulting from the combination of tools. For example, a designer may create a satisfactory site proposal quickly in Blocktype, but then want to combine this with a local demographic study in ArcGIS. This is where interoperability platforms come in, unlocking new functionality through workflows combining multiple tools. Further, these can enable future integration of useful functionality from as yet unidentified or uncreated tools. It therefore seems reasonable that an interoperability platform should form the backbone of any urban planning workflow. Of the two options considered here, Speckle has more plugins for AEC tools, as well as a version control system for collaborative modelling, so it is proposed for future selection.

Videogame engines have as yet only been utilised in urban planning for visualisation. However, their potential for calculation means they could be applied to a wider range of aspects of this field. With UE5’s lack of functionality oriented towards urban planning it is difficult to justify its use over an established tool, but the city simulation of Cities Skylines II entails a rich modelling environment. When partnered with its intentionally user-friendly UI design and the scope of its modifiability for adding any additional functionality - such as citizen participation features, parametric modelling, and generative design - it presents an intriguing prospect for an urban design tool in its own right. However, fully realising its possibilities does require further research due to its present lack of application to real-life urban planning.

If Cities Skylines II is to form a core part of the overall workflow, then geospatial data import requires further consideration as most established tools contain it by default whereas this does not. They most commonly use OpenStreetMap, as it is free, but MapBox provides OpenStreetMap with additional useful data - and its lack of a UI is then mitigated by use of Cities Skylines II as the interface. However, Giraffe already integrates MapBox and its data into a tool with additional functionality, including a highly customisable conceptual analysis calculation system and parametric modelling features. It also has a free tier and a Speckle extension. One limitation of this workflow would be its lack of a dedicated GIS tool capable of complex analyses. However, between Giraffe and Cities Skylines II’s built-in calculations, and the modifiability of the latter in adding more, this functionality can still be included in the approach.

## Conclusion

It is the citizens who understand best the areas where they live, but planners have a level of technical education which they lack. This research has reviewed the software available to the planning industry with the intent of utilising it to bridge this knowledge gap to enable better public participation in planning.

Surveying the GIS software underlined the need for tools that are computationally powerful and simple to use. Some potential resolutions to this were parametric modelling and generative design, found amongst the RCGA applications. Interoperability platforms were then identified to ease connections between different tools and data. Finally, the potential of videogame engines was explored, and Cities Skylines II emerged with a promising combination of a user-friendly UI and computationally powerful simulation.

The resulting proposed workflow crystallises Cities Skylines II as a directly integrable addition to the urban planning toolkit, with its core set of features that can be directly leveraged to bridge the technical knowledge gap between communities and professional planners, enabling more meaningful public participation in planning.

## Future work

Cities Skylines II would be experiencing a new application in the workflow as described above. Further research is therefore needed to identify the specifics of how this would be accomplished, including developing a connector for Speckle and programming modifications for parametric modelling and generative design. The workflow as a whole would then require testing, especially with communities to assess its effectiveness in improving public participation.

The tools examined in this research provide other functionality not discussed in detail here. Further work may therefore explore a different analysis paradigm, and thus could reasonably produce a different schema and highlight different functionality.

This research has focussed on eliminating participatory barriers created by current principles of software design, but does not account for communities who are fundamentally unwilling or unable to engage with software as a concept. Further work is thus required to ensure that citizens with limited educational background, low digital literacy, and disabilities are empowered to engage with the process, with any real-world testing of the proposed workflow needing to account for this.

## Data Availability

All data generated or analysed during this study are included in this published article.
